# Health-related quality of life and caregiving burden in pediatric clubfoot patients treated with Ponseti method in China: a cross-sectional study

**DOI:** 10.1186/s12891-026-10075-w

**Published:** 2026-06-12

**Authors:** Yi Yang, Xuan Yang, Lejing Guan, Jingfang Xu, Wenhao Chen, Guannan Bai

**Affiliations:** 1 Department of Orthopedics, Children’s Hospital, Zhejiang University School of Medicine, National Clinical Research Center for Children and Adolescents’ Health and Diseases, Hangzhou, Zhejiang Province People’s Republic of China; 2 Department of Pediatric Orthopedics, Xinhua Hospital Affiliated to Shanghai Jiaotong University School of Medicine, Shanghai, People’s Republic of China; 3 Department of Child Health Care, Children’s Hospital, Zhejiang University School of Medicine, National Clinical Research Center for Children and Adolescents’ Health and Diseases, Hangzhou, Zhejiang Province People’s Republic of China

**Keywords:** Clubfoot, Ponseti method, HRQoL, PedsQL GCM, PedsQL FIM, Caregiving burden

## Abstract

**Background:**

Clubfoot is a common congenital foot deformity, typically managed with the standard Ponseti method. However, the impact of clubfoot on the quality of life for children and their caregivers in China remains largely unexplored. This study aimed to evaluate the HRQoL of clubfoot patients and their parental caregivers as well as their caregiving burden, while identifying key factors influencing these outcomes.

**Methods:**

An online survey was conducted in WeChat groups for children born with clubfoot and treated with Ponseti method. The custom questionnaire including general and clinical information of patients and their caregivers, caregiver-proxy-reported HRQoL of patients (PedsQL™ 4.0 GCM), HRQoL of caregivers (PedsQL™ 3.0 FIM) and the caregiving burden (ZBI-12) were collected. The HRQoL of patients were compared with healthy reference and the HRQoL of caregivers and ZBI were compared across different age groups. Pearson analyses were made to explore their correlation and multivariate linear regression models were used to identify their associate factors. All statistical analyses were performed using SPSS version 27.0 (*p <* 0.05).

**Results:**

A total of 180 cases were included in this study. Compared to healthy reference, PedsQL GCM showed a significant decrease in physical functioning and social functioning subscales in the 0-1 yr group but a significant increase in every scale score and total score in the 5-18yrs group. The lowest PedsQL FIM was observed in the 1-2yrs group and the highest in the 5-18yrs group. All PedsQL GCM scale scores and the total scores correlated significantly with PedsQL FIM scores in the 2-18yrs group. ZBI scores gradually increased during treatment and dropped after 5yrs. However, 54.44% of the caregivers included reported no to little burden. The factors associated with patients’ HRQoL included age and treatment-related complications, and the factors associated with caregivers’ HRQoL were PedsQL GCM, ZBI and brace adherence.

**Conclusion:**

Children with clubfoot treated with the standard Ponseti method can achieve excellent quality of life after the early stage of treatment. Preventing treatment-related complications can improve patients’ HRQoL. Clinicians should reinforce brace adherence education during follow-ups to enhance caregivers’ HRQoL. Despite the protracted treatment, the caregiving burden is acceptable for most families. These findings can provide valuable insights for caregiver education regarding this condition.

**Trail registration:**

Not applicable.

**Supplementary Information:**

The online version contains supplementary material available at 10.1186/s12891-026-10075-w.

## Introduction

Idiopathic Clubfoot, also known as Congenital talipes equinovarus, is a prevalent congenital musculoskeletal deformity, occurring in approximately 1 to 2 per 1000 live births worldwide [1]. Its exact etiology is unclear, though genetic predisposition and environmental factors are implicated [2, 3]. The deformity is characterized by forefoot adduction, midfoot cavus, hindfoot varus and ankle equinus, leading to significant functional impairment if left untreated [4]. The Ponseti method is the most widely adopted treatment involving weekly casting, Achilles tenotomy and bracing with foot abduction orthoses for at least 4 years [5, 6]. While numerous studies have demonstrated the successful clinical outcomes of the Ponseti method, the quality of life (QoL) of patients during this prolonged treatment has received limited attention [7–11]. Persistent treatment may affect the gross motor skills and neurology development and reduce daily activities of children [12, 13]. Treatment-related complications like cast slippage, skin rashes, pain, pressure sores and infection, which potentially lead to delay successful treatment or incomplete treatment, negatively impact the health status of affected children and their caregivers [14, 15]. Additionally, caregivers are subjected to medical, social, financial, and emotional strain [16]. Accepting the condition and managing long-term treatment can be psychosocially demanding [17]. These factors collectively impair caregivers’ QoL and increase their caregiving burden.

Health-related quality of life (HRQoL) is a specific dimension of overall QoL that focuses on how an individual's health status and medical condition impact their perceived well-being and ability to function in daily life [18]. Various instruments have been employed to assess HRQoL correlates, including health risks and conditions, functional status, social support, and socioeconomic status [19]. To date, few studies have explored HRQoL of clubfoot patients. Vahidi [20] and Seidel [21] used generic instrument of PedsQL to assess QoL in clubfoot patients and reported favorable results after Ponseti treatment. Svehilk adapted disease-specific instruments (FRS [22], ICFSG questionnaire [23], AOFAS questionnaire [24], SF-36 [25]) to evaluate long-term outcomes in clubfoot patients treated by different methods, finding better QoL in the Ponseti group compared to the surgical group [26]. Conversely, Wallander, utilizing SF-36 and EQ-5D [27], reported impaired activities of daily life after a 64-year follow-up [28]. Bessellaar found significantly lower scores on subscales of motor functioning, sleep, lung and skin problems during Ponseti bracing [29]. Previous studies investigating the HRQoL of clubfoot patients have yielded various results. This inconsistency is likely attributable to variations in study methodology, sample cohorts, treatment modalities, and treatment stages. To our knowledge, published literature on HRQoL of clubfoot patients in Chinese population appears to be absent. Moreover, most studies have focused on the physical and psychosocial consequences for patients, with limited attention paid to their caregivers' conditions.

There remains a notable gap in understanding the influence of clubfoot on HRQoL of patients across different treating stages. The psychosocial and functional burden experienced by their primary caregivers have been less comprehensively explored. Therefore, this study aims to bridge this gap by collecting HRQoL data of clubfoot patients and their caregivers from the Chinese population treated with the standard Ponseti method. By quantifying the specific domains of HRQoL deficits and caregiving burden, our findings will provide an evidence-based foundation for developing more holistic and patient-centered care strategies. In China, an increasing number of physicians are using social messaging apps to maintain contact with patients. This study employed WeChat, a widely used platform in China, to conduct online surveys, facilitating more convenient and efficient data collection. The primary aim was to assess the HRQoL of children with clubfoot and their caregivers and their correlation. The secondary aim was to measure the caregiving burden. The third aim was to identify associated factors affecting the HRQoL of patients and their caregivers.

## Methods

### Study design and participants

This online survey was conducted between March 1, 2025, and April 15, 2025. Participants were recruited from online WeChat groups for clubfoot patients. WeChat is a widely used instant messaging application in China, comparable to WhatsApp in its functionality. In China, physicians commonly utilize the WeChat to enhance patient engagement, facilitate follow-up care, and obtain timely feedback. In our study, participating physicians established WeChat groups for the caregivers of pediatric clubfoot patients who had undergone treatment using the standard Ponseti method. These physician-administered WeChat groups offer functions such as outpatient appointment scheduling and provide a valuable platform for patients to interact and receive mutual support. Membership in these groups was exclusively limited to caregivers of clubfoot children who had been treated by the physician. The physicians distributed the electronic questionnaire within the WeChat groups. Primary parental caregivers with direct knowledge of the child’s health were invited to participate and complete the questionnaire. Inclusion criteria were: (1) caregivers of patients diagnosed with clubfoot and treated with the standard Ponseti method, and (2) patients aged 0 to 18 years. Exclusion criteria were: (1) caregivers unwilling or unable to complete the electronic questionnaire; (2) children with positional clubfoot; (3) children with atypical or neuromuscular clubfoot associated with other anomalies. The study was approved by the Medical Ethics Committee of the Children’s Hospital, Zhejiang University School of Medicine (2025-IRB-0161-P-01) and it adhered to the Declaration of Helsinki. All parents provided electronically signed consent prior to completing the questionnaire.

### Treatment protocol

Clinicians strictly adhered to the standard Ponseti method in clinical procedures [6]. Each patient underwent serial casting to gradually correct adduction and varus deformities. Percutaneous Achilles tenotomy was then performed for patients lacking sufficient ankle dorsiflexion. A subsequent casting was applied for three weeks, followed by orthotic brace immobilization. Bracing was initially prescribed for 23 h daily for the first three months. This was gradually reduced to 16 h at one year of age to accommodate gross motor development. As patients’ ambulation needs increased, bracing time was further reduced to 12 h until five years of age. Follow-up appointments were scheduled every three months. The decision to discontinue treatment was based on the final clinical appearance and adequate ankle dorsiflexion. Relapse clubfoot were defined as the reappearance of any typical deformity that needs any further treatment after the initial correction [30]. Relapsed cases necessitated repeated casting and bracing, or muscle balancing and bony surgical procedures.

### Data collection

A custom questionnaire was developed through a literature review and expert consensus. Five pediatric orthopedic specialists and psychosociology researchers contributed to its development. Through three rounds of discussion, they refined the structured questionnaire, which included sections on patient and family general characteristics, HRQoL of patient and caregiver, and caregiving burden. A research assistant created the online version of the questionnaire using Wenjuanxing (https://www.wjx.cn/), a widely used platform for online surveys in China. A QR code was generated to facilitate completion by caregivers via mobile phones. A pilot study involving 15 caregivers was conducted to assess question logic, clarity, and ease of completion. Based on their feedback, the questionnaire underwent further revisions. The final version was distributed through WeChat communities starting March 1, 2025. Caregivers could direct questions to the research coordinator (YY and XY) within WeChat. Two research assistants (LG and GB) reviewed and verified completed questionnaires weekly to ensure data integrity.

General information gathered through the questionnaire included age, gender, only child status, residence, marital status of caregiver, educational level of parents, annual family income, treatment costs and health insurance coverage. Clinical variables, gathered through medical records, encompassed laterality, primary Pirani score, treatment initiation time, treatment-related complications, brace adherence, Achilles tenotomy, and relapse.

### Measurements

The HRQoL for both patients and their caregivers were assessed using proxy-reported versions of the PedsQL™ 4.0 Generic Core Module (GCM) and the PedsQL™ 3.0 Family Impact Module (FIM). The Chinese versions of both instruments have demonstrated good reliability and validity [31, 32]. For children younger than 2 years, the PedsQL Infant and Toddler Instrument was utilized. This instrument comprises five scales: physical functioning, physical symptoms, emotional functioning, social functioning and cognitive functioning. For children aged 2–18 years, the PedsQL GCM was employed, encompassing five scales: physical functioning, emotional functioning, social functioning, school functioning and psychosocial health summary. The PedsQL FIM consists of 28 items across 6 domains: physical functioning (6 items), emotional functioning (5 items), social functioning (4 items), cognition functioning (4 items), communication (3 items), worry (5 items), daily activity (3 items) and family relationships (5 items). The recall period for all measures was the past month. In the present study, the Cronbach’s alpha coefficients of PedsQL Infant and Toddler Instrument are 0.96 for children aged 0–1 years and 0.97 for children aged 1–2 years. For the PedsQL GCM, coefficients were 0.97 (children aged 2–5 years), 0.92 (children aged 5–8 years), and 0.96 (children aged 8–18 years). Cronbach’s alpha coefficients for individual scales of the PedsQL Infant and Toddler Instrument and PedsQL GCM are presented in Supplementary Table S1. The overall Cronbach’s alpha for the PedsQL FIM was 0.98, with individual scale coefficients detailed in Supplementary Table S2. The scoring algorithms for these instruments are similar. Response choices ranged from 0 to 4 (0 = never a problem, 1 = seldom a problem, 2 = sometimes a problem, 3 = often a problem, 4 = always a problem). Item scores were linearly and reversely transformed to a 0–100 scale (0 = 100, 1 = 75, 2 = 50, 3 = 25, 4 = 0). Scale and total scores were calculated as the sum of item scores divided by the number of answered items. Higher scores indicate better HRQoL. Caregiving burden was assessed by using short version of Zarit Burden Interview (ZBI-12) [33]. The ZBI-12 is a widely used instrument measuring a caregiver’s subjective burden, reflecting their perception of how caring for another affects their personal life, including aspects of relationship burden, emotional well-being, social and family life, finances, and loss of control [34]. The Chinese version shows good reliability and validity [35, 36]. It measures the caregiving burden with a total score range from 0 to 48, with 0–4 points awarded per item. Higher total scores indicate heavier burden. Caregiving burden was categorized into three levels based on the total ZBI score: no to little burden (0–10), mild to moderate burden (10–20), and high burden (20–48).

### Statistical analyses

All statistical analyses were performed using SPSS version 27.0 (SPSS Inc., Chicago, USA). Descriptive statistics were calculated, including means ($\overline{x }$)and standard deviations (SDs) for normally distributed continuous variables, and medians (M)with interquartile ranges (IQR) for non-normally distributed continuous data. Categorical variables were presented as numbers and percentages. Next, we compared the mean total and scale scores of patients’ HRQoL with healthy reference values derived from published literature [37–39]. We also compared the mean total and scale scores of the PedsQL FIM and ZBI across different age groups. Pearson correlation analysis was used to evaluate associations between variables. Furthermore, independent samples t-tests and one-way analysis of variance (ANOVA) were employed to assess differences in mean scale and total scores of the PedsQL Infant and Toddler Instrument, PedsQL GCM, and PedsQL FIM across subgroups defined by general and clinical characteristics. Finally, to identify factors associated with the total HRQoL scores for both patients and caregivers, variables with statistical significance were selected and included in multivariate linear regression models.

Statistical significance was set at *p <* 0.05. For Pearson correlation analysis, correlations were classified as high (*r ≥* 0.5), moderate (0.3 ≤ *r <* 0.5), and low (*r <* 0.3) [40]. In addition to statistical significance, effect sizes, specifically Cohen’s d and partial eta squared (${\upeta}_{p}^{2}$), were calculated to assess clinical relevance. Regarding differences between two groups, Cohen’s d was interpreted as follows: 0.2 ≤ d < 0.5 indicated a small difference; 0.5 ≤ d < 0.8 indicated a moderate difference; and d ≥ 0.8 indicated a large difference [41]. For differences across three or more groups, ${\upeta}_{p}^{2}$ was used and interpreted as follows: 0.01 ≤ ${\upeta}_{p}^{2}$< 0.06 indicated a small difference; 0.06 ≤ ${\upeta}_{p}^{2}$ < 0.14 indicated a moderate difference; and ${\upeta}_{p}^{2}$≥ 0.14 indicated a large difference.

## Results

A total of 206 questionnaires were initially collected. After applying inclusion/exclusion criteria, five participants declined participation, six cases of postural clubfoot, and 15 cases with neurogenic or other associated anomalies were excluded. These excluded cases included: 8 with arthrogryposis, 2 with Duchenne muscular dystrophy, 3 with tethered cord syndrome, 1 with myelomeningocele, and 1 with Charcot-Marie-Tooth disease. Ultimately, 180 patients were enrolled in the study. The general and clinical characteristics of the enrolled patients are summarized in Tables 1 and 2, respectively. All patients initiated treatment within three months after birth and had commenced the bracing phase. Based on the age-specific dimensions of the PedsQL scales and variations in the treatment protocol, patients were divided into four age groups: 0–1 year, 1–2 years, 2–5 years, and 5–18 years.

**Table 1:** General characteristics of cases (*n =* 180)

Variables	Values*
Age (year) [median (interquartile range)]	3.90 (2.10, 6.80)
Age groups	
0–1 years	24 (13.33)
1–2 years	25 (13.89)
2–5 years	50 (27.78)
5–18 years	81 (45.00)
Gender	
Boy	132 (73.33)
Girl	48 (26.67)
Only child	
Yes	89 (49.44)
No	91 (50.56)
Residence	
Cities	106 (58.89)
Rural areas	74 (41.11)
Marital status	
Married	175 (97.22)
Unmarried or divorced	5 (2.78)
The highest education level of the father	
Low	34 (18.89)
Medium	45 (25.00)
High	101 (56.11)
The highest education level of the mother	
Low	39 (21.67)
Medium	32 (17.78)
High	109 (60.55)
Annual family income (RMB)	
< 100,000	73 (40.56)
100,000- 200,000	59 (32.78)
> 200,000	48 (26.66)
Cost related to clubfoot treatment in total (RMB)	
< 30,000	62 (34.44)
30,000–50,000	63 (35.00)
> 50,000	55 (30.56)
Medical Insurance	
Yes	122 (67.8)
No	58 (32.2)
Perceived financial burden	
Yes	122 (67.78)
No	68 (32.22)
Which cost do you think brings economic burden?(multiple choices)	
Casting	47 (26.11)
Orthopedic brace	89 (49.44)
Transportation and accommodation expenses	71 (39.44)
Drug	14 (7.78)
Others	11 (6.11)

^*^This table presents numbers, percentages, median, and interquartile range. Regarding the educational level, low means no education, primary school or middle school; middle level means high school, technical school, vocational secondary school, and vocational high school; high level means bachelor’s degree or above according to the Chinese Standard Classification of Education

**Table 2:** Clinical characteristics of cases (*n =* 180)

Variables	Values*
Laterality	
Unilateral	105 (58.33)
Bilateral	75 (41.67)
Primary Pirani scores	
≤ 5.0	24 (13.33)
5.5	66 (36.67)
6.0	90 (50.00)
Treatment initiation time	
Within one week of birth	31 (17.22)
Within half a month of birth	51 (28.33)
Within one month of birth	35 (19.45)
Within three months of birth	63 (35.00)
Treatment-related complications	
No	117 (65.00)
Yes	63 (35.00)
What kind of complications have occurred?(multiple choices)	
Swelling	54 (30.00)
Cast slippage	24 (13.33)
Rashes	19 (10.56)
Pain	11 (6.11)
Pressure sores	7 (3.89)
Abnormal vascularity	3 (1.67)
Infection	1 (0.56)
Others	5 (2.78)
Brace adherence	
All completion (> 90% of the acquired time)	64 (35.56)
Most completion (90%−75% of the acquired time)	95 (52.78)
Partially completion (75%−50% of the acquired time)	19 (10.56)
Low completion (< 50% of the acquired time)	2 (1.11)
Achilles tenotomy	
Yes	170 (94.44)
No	10 (5.56)
Relapse	
Yes	11 (6.11)
No	169 (93.89)

^*^This table presents numbers and percentages

### HRQoL in patients

Table 3 presents the PedsQL total scores and scale scores of clubfoot patients compared to those of healthy children across all age groups. A significant reduction in physical functioning and social functioning scores was observed in the 0–1 year group (*p <* 0.05). In contrast, no significant differences in total or scale scores were observed in the 1–2 and 2–5 years groups. However, for children aged 5–18 years, both total and scale scores showed a significant increase compared to the healthy reference group (*p <* 0.05).

**Table 3:** PedsQL 4.0 GCM scale scores and total scores comparisons between healthy references and children with clubfoot

	Healthy^a^ (*n =* 246)	0–1 years (*n =* 24)	*p*	Healthy^b^ (*n =* 141)	1–2 years (*n =* 25)	p	Healthy^c^ (*n =* 36)	2–5 years (*n =* 50)	*p*	Healthy^d^ (*n =* 282)	5–18 years (*n =* 81)	*p*
Physical functioning	87.54 (11.16)	78.30 (17.46)	0.00	90.32 (8.96)	86.78 (17.54)	0.22	81.25 (11.60)	83.31 (23.45)	0.62	76.57 (13.78)	86.64 (19.11)	0.00
Physical symptoms	83.45 (10.39)	79.27 (14.02)	0.09	87.54 (9.29)	85.30 (14.80)	0.34	/	/	/	/	/	/
Emotional functioning	76.59 (13.71)	76.82 (16.72)	0.94	78.60 (12.80)	77.42 (17.78)	0.69	72.36 (14.66)	79.00 (27.26)	0.19	76.60 (17.63)	86.25 (19.75)	0.00
Social functioning	89.62 (14.87)	82.29 (19.82)	0.02	91.14 (10.77)	90.80 (14.12)	0.89	82.64 (13.44)	86.00 (23.04)	0.42	81.18 (15.71)	90.31 (16.75)	0.00
Cognitive functioning	83.11 (20.65)	79.17 (19.91)	0.35	84.65 (15.76)	86.33 (19.95)	0.65	/	/	/	/	/	/
School functioning	/	/	/	/	/	/	76.62 (14.34)	80.17 (32.55)	0.53	77.52 (17.40)	84.25 (16.73)	0.00
Psychosocial Health Summary Score	/	/	/	/	/	/	77.30 (10.90)	81.88 (23.28)	0.29	80.13 (14.66)	86.71 (14.90)	0.00
Total scores	82.47 (9.95)	78.62 (13.89)	0.05	85.55 (8.74)	84.31 (14.69)	0.57	78.80 (9.70)	82.43 (22.46)	0.34	82.38 (13.29)	86.68 (15.58)	0.02

The values are presented as mean (standard deviation)

^a,b^Healthy reference values for children aged 0–1 years and 1–2 years are cited from the study by Varni et al. [39]

^c^Healthy reference values for children aged 2–5 years are cited from the study by Chan et al. [37]

^d^Healthy reference values for children aged ≥ 5 years are cited from the study by Hao et al. [38]

### HRQoL in caregivers

Table 4 presents the PedsQL FIM total and scale scores. The lowest total score was observed in the 1–2 years group (M:72.92, IQR: 61.11–91.67). In contrast, the highest total score was found in the 5–18 years group (M:94.44, IQR: 72.92–100.00). This difference between age groups was statistically significant (*p =* 0.01). Regarding scale scores, significant differences among groups were identified in the physical functioning and worry scales. The post hoc analysis in supplementary table S3 showed significantly lower score in the worry scale for 0–1 year group (M: 70.00, IQR: 50.00–81.25) and in the physical functioning scale for the 1–2 years group (M: 58.33, IQR: 54.17–100.00), when compared to the 5 −18 years group.

**Table 4:** PedsQL 3.0 FIM total scores and scale scores and ZBI for children with clubfoot (*n =* 180)*

		0–1 years (*n =* 24)	1–2 years (*n =* 25)	2–5 years (*n =* 50)	5–18 years (*n =* 81)	*p*
FIM	Physical functioning	75.00 (61.46,92.71)	58.33 (54.17,100.00)	81.25 (50.00,100.00)	100.00 (70.83,100.00)	0.01
	Emotional functioning	77.50 (68.75,96.25)	75.00 (55.00,100.00)	82.50 (55.00,100.00)	100.00 (70.00,100.00)	0.06
	Social functioning	81.25 (75.00,100.00)	75.00 (62.50,100.00)	100.00 (65.62,100.00)	100.00 (75.00,100.00)	0.13
	Cognitive functioning	75.00 (63.75,100.00)	75.00 (55.00,100.00)	90.00 (70.00,100.00)	100.00 (75.00,100.00)	0.15
	Communication	79.17 (75.00,100.00)	100.00 (66.67,100.00)	100.00 (58.33,100.00)	100.00 (75.00,100.00)	0.11
	Worry	70.00 (50.00,81.25)	70.00 (50.00,90.00)	70.00 (50.00,100.00)	90.00 (70.00,100.00)	0.01
	Daily Activities	79.17 (64.58,100.00)	75.00 (50.00,100.00)	100.00 (50.00,100.00)	100.00 (75.00,100.00)	0.05
	Family Relationships	87.50 (75.00,100.00)	100.00 (75.00,100.00)	100.00 (50.00,100.00)	100.00 (75.00,100.00)	0.11
	Total scores	75.00 (63.89,91.49)	72.92 (61.11,91.67)	81.94 (59.20,98.61)	94.44 (72.92,100.00)	0.01
ZBI	Total scores	9.00 (3.75, 11.25)	12.00 (9.00, 19.00)	14.00 (6.00, 21.75)	8.00 (4.00, 15.00)	0.01

^*^Given that the values were not normally distributed after grouping, we adopted nonparametric statistical methods. The values are presented as median (interquartile range)

### Caregiving burden

**Fig. 1:**
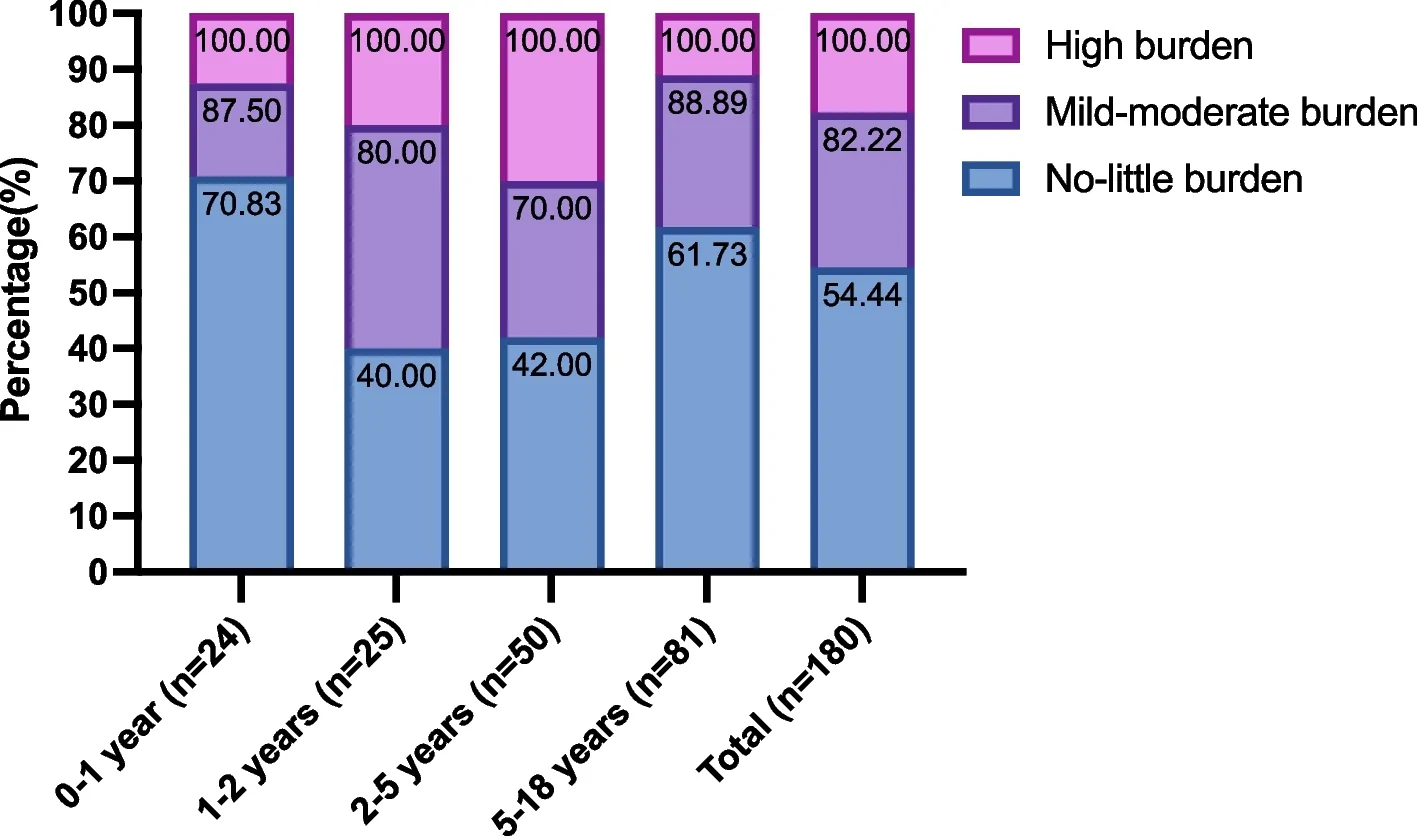
Distribution of burden levels across different age groups

Table 4 shows that ZBI scores gradually increased throughout the treatment process, rising from 9.00 to 14.00, before a dramatic drop to 8.00. The lowest burden was observed in the 5–18 years group, while the highest burden was in the 2–5 years group. Fig. 1 illustrates the distribution of burden levels across different age groups. Similarly, the proportion of families reporting no to little burden decreased during treatment, while the proportion reporting high burden increased from 12.50% to 30.00%, subsequently dropping to 11.11% after 5 years of age. Overall, more than half of the families reported no to little burden, and only 17.78% reported high burden.

### Correlation between HRQoL in patients and caregivers

Pearson correlation analysis between PedsQL GCM and PedsQL FIM scores for children younger than 2 years is presented in Supplementary Table S4. Only total score and emotional functioning scale score of PedsQL GCM showed significantly moderate to strong correlation with scale scores and total score of the PedsQL FIM. In Supplementary Table S5, for children aged 2–18 years, the correlation between PedsQL GCM and PedsQL FIM showed a strong correlation across total score and every scale score.

### Factors associated with PedsQL GCM and PedsQL FIM

Supplementary Table S6 comprehensively illustrates the differences in PedsQL GCM and PedsQL FIM total scores across general and clinical subgroups. Patients with characteristics such as having extra caregivers, residing in a city, possessing a higher parental educational level, having a higher family income, experiencing no perceived financial burden, having fewer complications, and reporting less caregiving burden exhibited significantly higher PedsQL GCM scores (*p <* 0.05). Similarly, caregivers residing in a city, with higher educational levels, higher family incomes, medical insurance coverage, no perceived financial burden, less caregiving burden, whose children experience fewer complications and higher brace adherence also showed significantly higher PedsQL FIM scores (*p <* 0.05). Effect sizes indicated small to large differences regarding clinical relevance. Surprisingly, a significantly higher PedsQL FIM score was observed in the subgroup with a higher primary Pirani score (*p =* 0.024, η^2^ = 0.04), which contradicts clinical observation. Table 5 presents the associated factors of PedsQL GCM and PedsQL FIM scores, calculated by multivariate linear regression analyses. Significant associated factors for PedsQL GCM scores were age (β: 0.89; 95% CI: 0.09, 1.69; *p =* 0.03) and treatment-related complications (β: −6.53; 95% CI: −11.61, −1.44; *p =* 0.01). For PedsQL FIM, significant associated factors included PedsQL GCM (β: 0.57; 95% CI: 0.44, 0.73; *p <* 0.001), ZBI (mild to moderate burden: β: −12.34; 95% CI: −16.88, −7.79; *p <* 0.001; high burden: β: −17.81; 95% CI: −23.64, −11.97; *p <* 0.001), and brace adherence (most completion: β: −5.80; 95% CI: −9.79, −1.80; *p =* 0.01; partial or low completion: β: −8.42; 95% CI: −14.51, −2.33; *p =* 0.01).

**Table 5:** Associated factors of total scores of PedsQL 4.0 GCM and PedsQL 3.0 FIM in the multivariate linear regression analyses (*n =* 180)

Variables	PedsQL GCM total scores		PedsQL FIM total scores	
	β (95% CI)	*p*	β (95% CI)	*p*
Age	0.89 (0.09 ~ 1.69)	0.03	0.59 (−0.04 ~ 1.23)	0.07
PedsQL GCM	/	/	0.57 (0.44, 0.73)	**<** 0.01
Gender				
Boy (*n =* 132)	Reference		Reference	
Girl (*n =* 48)	−1.75 (−7.40 ~ 3.91)	0.55	2.0 (−2.34 ~ 6.34)	0.36
Treatment-related complications				
No (*n =* 117)	Reference		Reference	
Yes (*n =* 63)	−6.53 (−11.61 ~ −1.44)	0.01	−2.77 (−6.79 ~ 1.25)	0.18
Only child				
Yes (*n =* 89)	Reference		/	/
No (*n =* 91)	−1.07 (−6.28 ~ 4.14)	0.69	/	/
Residence				
Cities (*n =* 106)	Reference		Reference	
Rural areas (*n =* 74)	−1.15 (−6.75 ~ 4.45)	0.69	1.19 (−3.14 ~ 5.51)	0.59
The highest education level of the mother				
High (*n =* 109)	Reference		Reference	
Medium (*n =* 32)	−8.34 (−16.74 ~ 0.07)	0.05	−0.62 (−7.10 ~ 5.86)	0.85
Low (*n =* 39)	−0.85 (−10.24 ~ 8.53)	0.86	−3.80 (−10.94 ~ 3.33)	0.29
The highest education level of the father				
High (*n =* 101)	Reference		Reference	
Medium (*n =* 45)	−5.18 (−12.46 ~ 2.09)	0.16	3.70 (−1.89 ~ 9.30)	0.19
Low (*n =* 34)	−9.05 (−18.65 ~ 0.54)	0.07	3.92 (−3.46 ~ 11.30)	0.30
Annual family income (RMB)				
> 200,000 (*n =* 48)	Reference		Reference	
100,000- 200,000 (*n =* 59)	−2.75 (−9.61 ~ 4.10)	0.43	−2.40 (−7.72 ~ 2.92)	0.37
< 100,000 (*n =* 73)	1.51 (−6.69 ~ 9.71)	0.72	−5.08 (−11.42 ~ 1.27)	0.12
Extra caregivers				
Yes (*n =* 117)	Reference		/	/
No (*n =* 63)	−5.26 (−10.91 ~ 0.38)	0.07	/	/
Insurance				
Yes (*n =* 122)	/	/	Reference	
No (*n =* 58)	/	/	−1.92 (−6.36 ~ 2.52)	0.40
Perceived financial burden				
No (*n =* 68)	Reference		Reference	
Yes (*n =* 112)	−3.30 (−9.22 ~ 2.62)	0.27	−4.21 (−8.87 ~ 0.46)	0.08
ZBI				
No to little burden(0–10) (*n =* 98)	/	/	Reference	
Mild to moderate burden (10–20) (*n =* 50)	/	/	−12.34 (−16.88 ~ −7.79)	**<** 0.01
High burden (> 20) (*n =* 32)	/	/	−17.81 (−23.64 ~ −11.97)	**< **0.01
Primary Pirani scores				
≤ 5 (*n =* 24)	/	/	Reference	
5.5 (*n =* 66)	/	/	1.26 (−4.70 ~ 7.22)	0.68
6.0 (*n =* 90)	/	/	2.91 (−2.99 ~ 8.80)	0.33
Brace adherence				
All completion (*n =* 64)	/	/	Reference	
Most completion (*n =* 95)	/	/	−5.80 (−9.79 ~ −1.80)	0.01
Partial completion or low completion (*n =* 21)	/	/	−8.42 (−14.51 ~ −2.33)	0.01
Adjusted R^2^	0.17		0.67	

## Discussion

Clubfoot, the most common congenital limb deformity, exhibits a higher prevalence in China than in European countries [42]. In recent years, government-promoted training programs for diagnosing and treating congenital structural deformities have led most families to receive timely, accurate advice from obstetricians, facilitating early intervention for affected infants [43]. The Ponseti method has revolutionized clubfoot management since its widespread adoption. Compared to earlier treatment modalities, it achieves excellent therapeutic outcomes with preserved foot function, establishing it as the current gold standard [26, 44]. Disease-specific instruments such as the OXAFQ-C questionnaire or PROMIS have been employed in the final functional assessment of clubfoot treatment [45, 46]. As a generic measure reflecting patients' and caregivers' physical, emotional, social, and psychosocial well-being, the PedsQL can assess their quality of life across different treatment stages. Our study, utilizing validated instruments of PedsQL infant and toddler, PedsQL GCM, PedsQL FIM in clubfoot patients and caregivers, offers several significant contributions. Firstly, to our knowledge, this is the first study to focus on the QoL of clubfoot patients across different age groups, detailing the impact of the prolonged treatment process. Secondly, this study comprehensively analyzes caregivers’ QoL and caregiving burden, providing a more holistic understanding of the disease's impact on families and characterizing the social profile of the affected population. Thirdly, our study is the first to identify factors associated with the HRQoL of clubfoot patients and caregivers. Furthermore, our investigation of a substantial sample of 180 cases from the Chinese population is representative of countries with a higher clubfoot prevalence.

### HRQoL in clubfoot patients and caregivers

This cross-sectional study comprehensively revealed changes in patients’ QoL throughout the treatment process. PedsQL scores for the 0—1 year age group showed a significantly decrease in the subscales of physical functioning and social functioning. Patients in this age group were in the early stage of treatment, requiring bracing for over 16 h per day. Their adaptation to bracing and prolonged immobilization likely contributed to this significantly impaired quality of life. However, this negative influence diminished as bracing time decreased. PedsQL scores for the 1–2 years and 2–5 years age groups showed comparable results to healthy references. The transition pattern revealed that the normalization of quality of life occurred after the first stage of treatment (0–1 year), preceding treatment cessation. This suggests that the use of braces in the mid-to-late stages of treatment (1–2 years, 2–4 years) had minimal impact on patients' quality of life. Our findings contradict those of Besselaar, who reported lower scores on physical functioning during bracing but a return to normal as bracing ended [29]. Besselaar broadly categorized cases into bracing and post-bracing groups, a methodology that may lead to inconsistencies. Our study employed a more refined grouping strategy, stratifying patients by the stages of required bracing time, thus illustrating more convincing results. Interestingly, after cessation of bracing, children in the 5–18 years age group exhibited significantly higher scores in each scale than the normal population. Since children's PedsQL scores were parent-proxy reported, we hypothesize that after experiencing the challenging treatment process, caregivers' expectations for the patients may decrease. Consequently, once their condition was largely resolved, caregivers' evaluation of the patients' quality of life may paradoxically increase. In general, our findings are in accordance with some previous studies [20, 21, 47], validating the effectiveness of the Ponseti method in preserving good long-term outcomes for treated children and reconfirming that children treated with the standard Ponseti method maintain excellent quality of life after treatment.

An impaired parental or familial QoL with congenital abnormalities has been previously validated [48]. Our study firstly adopted PedsQL FIM to investigate the HRQoL of clubfoot caregivers and found a notable decline in total score and physical functioning scale score in the 1- 2 years group. A possible explanation for this result may relate to bracing adherence. Firstly, as patients grew up and entered walking stage, they may have less tolerance for prolonged orthotic bracing. This requires more energy from caregivers in assisting and supervising brace wearing, thereby impacting their physical well-being. Secondly, parental resistance to bracing may arise as children show delays in gross motor skill milestone achievement, such as standing and walking [49]. A high percentage of gross motor deviation has been observed in children with clubfoot [50]. Garcia reported a delay in gross motor development at 9–12 months of age, finding that children with clubfoot would walk later than typically expected [51]. The temporary delay may evoke parental concern over long-term immobilization, leading to a drop in brace adherence. However, this transient decline in PedsQL FIM improved with the reduced bracing time after 1 year of age and the restoration of gross motor function of patients. Additionally, the PedsQL FIM for the 0–1 year age group showed a significantly lower score in the "Worry" scale, indicating that concern for the patient's illness is the primary factor affecting caregiver quality of life in the early stage of treatment. Ozdemir used DASS-21 questionnaire and found a more anxious and stressful status in parents of clubfoot patients at the beginning of treatment [17]. Agarwa prospectively evaluated the QoL in mothers of clubfoot patients during Ponseti treatment using the WHOQOL-BREF instrument and also found significant lower QoL in affected mothers, particularly in the domains of psychological health and environment. However, subsequent results across various stages of Ponseti treatment remained comparable [16]. Our results correspond to this, as scores in the “worry” scale for the other groups remained relatively low but comparable to post-treatment levels.

### Caregiving burden

The ZBI is a widely recognized and validated self-report questionnaire designed to measure the subjective burden experienced by caregivers [52]. While initially developed for caregivers of adults with dementia, its application has expanded significantly and proven highly valuable in assessing the burden faced by parents or guardians of children with chronic illnesses, disabilities, or complex medical needs [53–55]. Unlike an isolated limb problem, the treatment for clubfoot extends over several years and the caregiving burden may vary across different treatment stages. For patients experiencing relapse or residual deformities, the caregiving burden associated with the condition can be further prolonged. This study is the first to utilize the ZBI questionnaire to assess caregiving burden specifically in the clubfoot population. Our study found that the peak of caregiving burden for clubfoot occurs in 2—5 years of age, with 30.00% of cases reporting high burden. This surge may be attributed to the cumulative strain and treatment costs associated with this extended period. While clubfoot is considered a curable condition, unlike dementia or rare diseases like Duchenne muscular dystrophy with lifelong implications where a plateau in caregiving burden might be expected [56, 57], our findings conversely show a substantial decline in the ZBI scores as treatment concludes. Pediatric illnesses generally require prolonged familial care and attention. For clubfoot, the protracted treatment duration necessitates considerable financial and energetic contributions from families. Despite these demands, importantly, more than half of the families reported their burden as no to little. These results offer encouragement to those involved in treatment and provide guidance for disease consultation.

### The associate factors of HRQoL in patients and caregivers

Our study fundamentally identified determinants among general and clinical characteristics associated with HRQoL in clubfoot patients and their caregivers. Age, by dictating the stage of treatment, directly affects patients’ HRQoL. Notably, treatment-related complications showed a pronounced association with PedsQL GCM. Although these factors explain only 17% of the variance in PedsQL GCM, these findings carry significant clinical implications for practitioners. Issues such as swelling, pain, pressure sores, and skin rashes associated with cast or brace application inflict additional suffering on patients. Repeated casting due to slippage or over-tightness further exacerbates the child's distress and increases family costs and burden. We strongly recommend ensuring qualified and secure cast application, providing proper instruction on brace wear, and implementing timely observation during treatment. These are the most direct and simple methods to avoid complications, thereby improving the quality of life for patients.

Despite observed discrepancies in the lowest HRQoL scores reported by patients and caregivers, a persistent significant association between them suggests an underlying interconnection. PedsQL GCM demonstrated significant correlation with the PedsQL FIM in just emotional functioning scale for children younger than 2 years, while its overall strong correlations in the 2–18 years group highlighted its significant influence on PedsQL FIM. Notably, the present study identified ZBI scores and brace adherence as pronounced determinants associated with PedsQL FIM. ZBI, a reflection of caregiving experience, serves as an indicator of behavioral and socioeconomic status. Caregivers reporting high burden consequently suffer impaired quality of life. This finding is supported by Loo's review of caregiving burden in Asia, which concluded that such burden is associated with poorer self-rated health and reduced quality of life [58]. Our study attributed a decline in PedsQL FIM to issues with brace adherence. Surprisingly, 88.33% of patients (159/180) reported completing all or most of the acquired bracing time. Brace adherence is now considered a predictor of relapse following Ponseti method treatment [59, 60]. Existing literature consistently demonstrates that poor brace compliance elevates the risk of relapse by 3–7 times, with reported poor compliance rates varying from 11 to 49% [61–64]. This substantial variability may stem from inconsistent definitions of poor compliance. In our study, we defined poor compliance as lacking over 25% of prescribed bracing time and the poor compliance rate (11.67%) was relatively low compare to that of others. We believe that these positive results were largely attributable to the follow-up strategy employed in our study. By maintaining close contact with clubfoot caregivers via widely accessible social messaging platforms, the physicians were able to enhance disease education regarding the importance of brace adherence. This approach enabled timely communication and resolution of any questions or difficulties arising during bracing and facilitated supervision for caregivers to ensure timely and high-quality brace wear. Moreover, the WeChat group, served as a peer-support hub for caregivers, fostered the sharing of practical experiences and deepened their understanding of the potential consequences of poor compliance, ultimately enhancing brace adherence. However, it should be noted that since these data was self-reported, there was a potential for recall bias especially regarding brace wearing durations. Such memory inaccuracies could influence the reliability of the results. Additionally, our results showed a lower relapse rate of 6.11% (11/180), which did not demonstrate a significant impact on PedsQL GCM or PedsQL FIM within this cohort. However, Gelfer [10] assessed the quality of life of clubfoot patients using the OxAFQ instrument and found that relapse (37%) led to poorer physical scores and was associated with poor clinical and QoL outcomes. As this is a cross-sectional study, our cohort primarily comprised patients in the early bracing phase, limiting the ability to capture long-term outcomes. Consequently, the final relapse rate could not be reliably determined due to the brief follow-up period. Therefore, prospective longitudinal cohorts are recommended to elucidate the effects of relapse on HRQoL in clubfoot patients and their caregivers.

The Pirani score is a widely accepted tool for classifying clubfoot severity [65], with higher scores indicating more rigid and severe deformities [66]. In our study, caregivers of patients with a Pirani score below 5.0 reported the lowest PedsQL FIM total scores. Conversely, the group with a score of 5.5 had the highest QoL scores. This finding appears counterintuitive to clinical expectations. More severe deformities typically necessitate more intensive treatment, such as frequent casting and aggressive manipulation, which may be associated with increased risks of complications and pain [66]. Our current results do not align with this expectation for several reasons. Firstly, sample bias may be a contributing factor. Only 24 patients (15.47% of the total) had scores below 5.0. Secondly, while adjacent Pirani scores differentiate deformity severity, these incremental differences may not significantly impact the overall QoL for patients and their caregivers. Previous research by Walter [67] utilized a self-designed questionnaire and found no correlation between parental distress and deformity severity, which may offer a potential explanation for our findings.

### Limitations

This study has the following limitations. Firstly, established Chinese reference data for PedsQL Infant and Toddler was unavailable at the time of this study. The absence of such comparisons compromises the accuracy in part of the results. Secondly, this study is an online questionnaire study conducted within the WeChat group, and several factors may contribute to selection bias. Some caregivers may be unwilling or unable to participate in the survey, leading to selection bias. Some caregivers may voluntarily leave the group due to unsuccessful treatment or relapse, which may affect the overall relapse rate of clubfoot treated at this institution. Additionally, patients who were not enrolled in the WeChat group prior to its establishment could not participate in this study, resulting in a relatively small sample size for long-term follow-up and potentially impacting the results. Thirdly, the cross-sectional design inherently limits our ability to establish causal inferences and analyze longitudinal changes across sequential treatment stages. Future research should consider multi-center collaborations to establish prospective longitudinal cohort studies with extended follow-up in the clubfoot population.

## Conclusion

Our study is the first to characterize the HRQoL of patients and caregivers, as well as the caregiving burden, within the Chinese clubfoot population. We identified determinants associated with HRQoL in both patients and their caregivers. Children with clubfoot treated with the standard Ponseti method achieved excellent HRQoL after the early treatment phase, comparable to healthy children. Preventing treatment-related complications emerged as a significant factor for improving patient HRQoL. During the later treatment stage, clinicians should emphasize brace adherence education to enhance caregivers' HRQoL. Despite the protracted duration of the Ponseti method, the caregiving burden associated with clubfoot appears acceptable for most families. These findings can provide valuable insights for caregiver education regarding this condition.

## Supplementary Information

Supplementary Material 1.

## Data Availability

The datasets used and analyzed during the current study are available from the corresponding author on reasonable request.

## Abbreviations

HRQoL: Health-related quality of life
QoL: Quality of life
FRS: Functional Rating System
ICFSG: International Clubfoot Study Group
AOFAS: American Orthopaedic Foot and Ankle Society
SF-36: Short Form 36 General Health
EQ-5D: EuroQol-5 Dimensions
PedsQL GCM: PedsQL™ 4.0 Generic Core Module
PedsQL FIM: PedsQL™ 3.0 Family Impact Module
ZBI: Zarit Burden Interview
OxAFQ: The Oxford Ankle Foot
PROMIS: Patient-reported Outcome Measurement Information System
WHOQOL-BRE: WHO Quality of Life Instrument, Short Form
