# Supplementary material for: Health-related quality of life and caregiving burden in pediatric clubfoot patients treated with Ponseti method in China: a cross-sectional study
Source: BMC Musculoskelet Disord. 2026 Jun 12;27:709. doi: 10.1186/s12891-026-10075-w (PMC13491802; doi:10.1186/s12891-026-10075-w)
Supplement: Supplementary file 1 [file 12891_2026_10075_MOESM1_ESM.docx]

**Supplementary**

Table S1. The Cronbach’s alpha coefficients for each domain and total score of PedsQL Infant and Toddler and PedsQL GCM

|  | Physical functioning | Physical symptoms | Emotional functioning | Social functioning | Cognitive functioning | School functioning | Total score |
| --- | --- | --- | --- | --- | --- | --- | --- |
| 0-1y | 0.86 | 0.85 | 0.94 | 0.90 | 0.94 | / | 0.96 |
| 1-2y | 0.95 | 0.89 | 0.92 | 0.90 | 0.95 | / | 0.97 |
| 2-5y | 0.94 | / | 0.94 | 0.94 | / | 0.96 | 0.97 |
| 5-8y | 0.81 | / | 0.88 | 0.87 | / | 0.79 | 0.92 |
| 8-18y | 0.90 | / | 0.89 | 0.92 | / | 0.86 | 0.96 |

Table S2. The Cronbach’s alpha coefficients for each domain and total score of PedsQL FIM

| Physical functioning | Emotional functioning | Social functioning | Cognitive functioning | Communication | Worry | Daily  activities | Family  relationships | Total score |
| --- | --- | --- | --- | --- | --- | --- | --- | --- |
| 0.94 | 0.96 | 0.89 | 0.96 | 0.93 | 0.94 | 0.95 | 0.98 | 0.98 |

Table S3 The post hoc test p-values for different age groups on the PedsQL FIM

|  | Physical functioning | Worry | Total score |
| --- | --- | --- | --- |
| 0-1 year vs. 1-2 years | 1.00 | 1.00 | 1.00 |
| 1-2 years vs. 2-4 years | 1.00 | 1.00 | 1.00 |
| 1-2 years vs. 5-18 years | **0.02** | 0.13 | **0.04** |
| 0-1year vs. 2-4 years | 1.00 | 1.00 | 1.00 |
| 0-1year vs. 5-18 years | 0.15 | **0.03** | 0.07 |
| 2-4 years vs. 5-18 years | 0.14 | 0.17 | 0.18 |

*Note*. vs. means versus. The values shown are adjusted p-value

Table S4. Correlation between PedsQL GCM total scores and scale scores and PedsQL FIM total scores and scale scores for children aged younger than 2 years (n=49).

|  | Physical Functioning | Emotional Functioning | Social Functioning | Cognitive Functioning | Communication | Worry | Daily Activities | Family Relationships | Total scores | ZBI scores |
| --- | --- | --- | --- | --- | --- | --- | --- | --- | --- | --- |
| Physical functioning | .41^**^ | .41^**^ | .41^**^ | .25 | .43^**^ | .43^**^ | .32^*^ | .40^**^ | .49^**^ | -.30* |
| Physical symptoms | .36^*^ | .43^**^ | .30^*^ | .37^**^ | .48^**^ | .33^*^ | .27 | .48^**^ | .50^**^ | -.14 |
| Emotional functioning | .56^**^ | .56^**^ | .59^**^ | .37^**^ | .55^**^ | .52^**^ | .43^**^ | .44^**^ | .65^**^ | -.40** |
| Social functioning | .41^**^ | .26 | .31^*^ | .21 | .39^**^ | .18 | .15 | .37^**^ | .38^**^ | -.22 |
| Cognitive functioning | .312^*^ | .27 | .33^*^ | .19 | .41^**^ | .12 | .09 | .22 | .33^*^ | -.13 |
| Total scores | .50^**^ | .49^**^ | .49^**^ | .35^*^ | .55^**^ | .41^**^ | .32^*^ | .46^**^ | .58^**^ | -.30* |

***p<0.05, **p<0.001**

Table S5. Correlation between PedsQL GCM total scores and scale scores and PedsQL FIM total scores and scale scores for children aged from 2 to 18 years (n=131).

|  | Physical Functioning | Emotional Functioning | Social Functioning | Cognitive Functioning | Worry | Communication | Daily Activities | Family Relationships | Total scores | ZBI scores |
| --- | --- | --- | --- | --- | --- | --- | --- | --- | --- | --- |
| Physical functioning | .55^**^ | .55^**^ | .64^**^ | .53^**^ | .55^**^ | .49^**^ | .54^**^ | .52^**^ | .61^**^ | -.45^**^ |
| Emotional functioning | .63^**^ | .66^**^ | .63^**^ | .61^**^ | .57^**^ | .57^**^ | .62^**^ | .53^**^ | .68^**^ | -.49^**^ |
| Social functioning | .55^**^ | .52^**^ | .65^**^ | .55^**^ | .54^**^ | .55^**^ | .52^**^ | .56^**^ | .62^**^ | -.51^**^ |
| School functioning | .63^**^ | .62^**^ | .66^**^ | .59^**^ | .54^**^ | .56^**^ | .56^**^ | .59^**^ | .67^**^ | -.53^**^ |
| Psychosocial Health Summary Score | .71^**^ | .71^**^ | .75^**^ | .69^**^ | .65^**^ | .65^**^ | .66^**^ | .65^**^ | .77^**^ | -.59^**^ |
| Total scores | .68^**^ | .67^**^ | .74^**^ | .66^**^ | .64^**^ | .61^**^ | .64^**^ | .63^**^ | .74^**^ | -.56^**^ |

***p<0.05, **p<0.001**

Table S6. Comparison of mean and standard deviation of PedsQL GCM and PedsQL FIM total scores (n=180).

|  | Factors | PedsQL GCM total scores | PedsQL FIM total scores |
| --- | --- | --- | --- |
|  | Gender |  |  |
|  | Boy (n=132) | 85.29 (17.19) | 81.64 (21.31) |
|  | Girl (n=48) | 80.99 (18.25) | 76.68 (18.80) |
|  | p value | 0.15 | 0.16 |
|  | Effect size* (Cohen’s d） | 0.24 | 0.25 |
|  | Only child status |  |  |
|  | Yes (n=89) | 86.78 (15.99) | 82.87 (18.87) |
|  | No (n=91) | 81.56 (18.64) | 77.82 (22.23) |
|  | p value | 0.05 | 0.10 |
|  | Effect size (Cohen’s d） | 0.30 | 0.25 |
|  | Extra caregivers |  |  |
|  | Yes (n=117) | 86.75 (15.88) | 82.51 (18.00) |
|  | No (n=63) | 79.30 (19.46) | 76.25 (24.69) |
|  | p value | **0.01** | 0.05 |
|  | Effect size (Cohen’s d） | 0.42 | 0.29 |
|  | Residence |  |  |
|  | Cities (n=106) | 86.44 ± 15.36 | 83.08 ± 20.07 |
|  | Rural areas (n=74) | 80.85 ± 19.89 | 76.36 ± 21.17 |
|  | p value | **0.04** | **0.03** |
|  | Effect size (Cohen’s d) | 0.32 | 0.33 |
|  | The highest education level of the father |  |  |
|  | Low (n=34) | 76.09 ± 20.42 | 73.02 ± 22.64 |
|  | Medium (n=45) | 79.12 ± 19.15 | 78.19 ± 20.64 |
|  | High (n=101) | 89.09 ± 13.89 | 83.72 ± 19.52 |
|  | p value | **<0.01** | **0.02** |
|  | Effect size ($\eta_{p}^{2}$) | 0.11 | 0.04 |
|  | The highest education level of mother |  |  |
|  | Low (n=39) | 79.18 ± 19.08 | 73.04 ± 21.18 |
|  | Medium (n=32) | 75.25 ± 21.77 | 75.67 ± 21.95 |
|  | High (n=109) | 88.53 ± 13.91 | 84.28 ± 19.36 |
|  | p value | **<0.01** | **0.01** |
|  | Effect size ($\eta_{p}^{2}$)) | 0.10 | 0.08 |
|  | Annual family income (RMB) |  |  |
|  | <100,000 (n=73) | 80.49 ± 20.76 | 74.59 ± 22.66 |
|  | 100,000- 200,000 (n=59) | 83.01 ± 16.39 | 79.91 ± 20.70 |
|  | >200,000 (n=48) | 91.09 ± 10.35 | 89.53 ± 13.57 |
|  | p value | **0.00** | **<0.01** |
|  | Effect size ($\eta_{p}^{2}$)) | 0.07 | 0.12 |
|  | Medical insurance |  |  |
|  | Yes (n=122) | 85.23 ± 16.30 | 82.93 ± 18.45 |
|  | No (n=58) | 81.85 ± 19.82 | 74.81 ± 24.12 |
|  | p value | 0.23 | **0.03** |
|  | Effect size (Cohen’s d) | 0.19 | 0.38 |
|  | Cost related to treatment(RMB) |  |  |
|  | <30,000 (n=62) | 85.36 (17.39) | 82.28 (19.71) |
|  | 30,000-50,000（n=63） | 83.99 (17.66) | 78.86 (22.49) |
|  | >50,000 (n=55) | 82.94 (17.77) | 79.77 (19.96) |
|  | p value | 0.76 | 0.64 |
|  | Effect size ($\eta_{p}^{2}$)） | 0.01 | 0.01 |
|  | Perceived financial burden |  |  |
|  | Yes (n=112) | 80.63 ± 19.21 | 74.33 ± 22.20 |
|  | No (n=68) | 89.92 ± 12.45 | 90.18 ± 13.18 |
|  | p value | **<0.01** | **<0.01** |
|  | Effect size (Cohen’s d) | 0.57 | 0.87 |
|  | Laterality |  |  |
|  | Unilateral (n=105) | 85.36 (18.70) | 79.94 (20.47) |
|  | Bilateral (n=75) | 83.72 (21.85) | 80.84 (21.23) |
|  | p value | 0.59 | 0.77 |
|  | Effect size (Cohen’s d） | 0.08 | 0.04 |
|  | Treatment initiation time |  |  |
|  | Within half a month of birth (n=82) | 83.53 (17.59) | 78.78 (21.01) |
|  | Within one month of birth (n=35) | 84.66 (18.60) | 81.87 (20.14) |
|  | within three months of birth (n=63) | 86.64 (17.10) | 81.46 (20.88) |
|  | p value | 0.91 | 0.66 |
|  | Effect size ($\eta_{p}^{2}$) | 0.01 | 0.01 |
|  | Primary pirani score |  |  |
|  | ≤5.0 (n=24) | 85.55 (15.47) | 69.73 (27.62) |
|  | 5.5 (n=66) | 85.92 (20.86) | 82.80 (17.47) |
|  | 6.0 (n=90) | 83.54 (20.60) | 81.32 (20.22) |
|  | p value | 0.75 | **0.02** |
|  | Effect size ($\eta_{p}^{2}$) | 0.002 | 0.04 |
|  | Treatment-related complications |  |  |
|  | Yes (n=63) | 79.74 (18.25) | 72.54 (21.12) |
|  | No (n=117) | 86.51 (16.73) | 84.50 (19.35) |
|  | p value | **0.01** | **<0.01** |
|  | Effect size (Cohen’s d） | 0.39 | 0.60 |
|  | Brace adherence |  |  |
|  | All completion (n=64) | 85.36 (17.35) | 85.19 (16.07) |
|  | Most completion (n=95) | 84.67 (16.38) | 79.39 (21.55) |
|  | Partial completion or low completion (n=21) | 78.06 (22.28) | 69.64 (25.53) |
|  | p value | 0.23 | **0.01** |
|  | Effect size ($\eta_{p}^{2}$) | 0.02 | 0.06 |
|  | Achilles tenotomy |  |  |
|  | Yes (n=10) | 84.80 ± 16.73 | 80.59 ± 20.60 |
|  | No (n=170) | 73.02 ± 26.69 | 75.69 ± 23.58 |
|  | p | 0.20 | 0.47 |
|  | Effect size | 0.67 | 0.24 |
|  | Relapse |  |  |
|  | Yes (n=11) | 81.01 (14.75) | 72.10 (24.98) |
|  | No (n=169) | 84.34 (17.71) | 80.85 (20.40) |
|  | p value | 0.54 | 0.18 |
|  | Effect size (Cohen’s d） | 0.20 | 0.40 |
|  | ZBI |  |  |
|  | No to little burden（0-10）(n=98) | 90.51 ± 12.38 | 91.50 ± 11.27 |
|  | Mild to moderate burden (10-20) (n=50) | 82.59 ± 14.15 | 73.15 ± 18.37 |
|  | High burden (>20) (n=32) | 67.05 ± 23.33 | 57.27 ± 22.92 |
|  | p value | **<0.01** | **<0.01** |
|  | Effect size ($\eta_{p}^{2}$) | 0.24 | 0.41 |

*Effect size: We calculated Cohen’s d for independent t-tests and partial eta squared (η_p_^2^) for ANOVAs to estimate the effect size. Based on benchmarks suggested by Cohen, effect sizes were indicated as small (d = 0.2, η_p_^2^ = 0.01), medium (d = 0.5, η_p_^2^ = 0.06), and large (d = 0.8, η_p_^2^ = 0.14) effects by Cohen’s d and η_p_^2^. p-value < 0.05. The values are presented as mean (standard deviation) for normally distributed values and as median (interquartile ranges) for non-normally distributed values.
